# Development and Comparative Evaluation of Two Highly Sensitive Immunosensor Platforms for Trace Determination of Copper Ions in Drinking Water Using a Monoclonal Antibody Specific to Copper-EDTA Complex

**DOI:** 10.3390/molecules28207017

**Published:** 2023-10-10

**Authors:** Ibrahim A. Darwish, Zongzhi Wang, Ryhan J. Darling

**Affiliations:** 1 Department of Pharmaceutical Chemistry, College of Pharmacy, King Saud University, Riyadh P.O. Box 2457 11451, Saudi Arabia; 2 State Key Laboratory of Freshwater Ecology and Biotechnology, Institute of Hydrobiology, Chinese Academy of Sciences, Wuhan 430072, China; 3 Department of Biochemistry, Medical College of Wisconsin, Milwaukee, WI 53226, USA

**Keywords:** copper ions, drinking water, immunoassays, ELISA, KinExA

## Abstract

This study describes the development of two highly sensitive immunosensor platforms for the trace determination of copper ions, Cu(II), in drinking water. These platforms were a microwell-based enzyme-linked immunosorbent assay (ELISA) and a kinetic exclusion assay (KinExA) with a KinExA^TM^ 3200 immunosensor. Both ELISA and KinExA were developed utilizing the same antibody and coating reagent. The antibody was a mouse monoclonal antibody, designated as 8D66, that specifically recognized Cu(II)-ethylenediamine tetraacetic acid complex (Cu(II)-EDTA) but did not recognize Cu(II)-free EDTA. The 8D66 monoclonal antibody was generated by the fusion of spleen cells of an immunized BALB/c mouse with SP2/0-Ag14 myeloma cells. The immunogen was a protein conjugate of Cu(II)-EDTA with keyhole limpet hemocyanin protein. The coating reagent was Cu(II)-EDTA covalently linked to bovine serum albumin protein (Cu(II)-EDTA-BSA). Both assays involved the competitive binding reaction between Cu(II)-EDTA complexes, formed in the sample solution, and Cu(II)-EDTA-BSA conjugate which has been immobilized onto ELISA plates (in ELISA) or polymethylmethacrylate beads (in KinExA) for a limited quantity of binding sites of the 8D66 antibody. In ELISA, color signals were generated by a peroxidase-labeled secondary antibody and 3,3′,5,5′-tetramethylbenzidine substrate. In KinExA, a fluorescein isothiocyanate-labeled secondary antibody was used to generate KinExAgram (trend-line fluorescence responses vs. time). The conditions of both ELISA and KinExA were investigated, and the optimum procedures were established. Both ELISA and KinExA were validated, and all validation parameters were acceptable. Many different metal ions that are commonly encountered in drinking water did not interfere with the Cu(II) analysis by both ELISA and KinExA. Both assays were applied to the determination of Cu(II) in drinking water with satisfactory accuracy and precision. Both assays were compared favorably with inductively coupled plasma atomic emission spectroscopy in terms of their abilities to accurately and precisely determine Cu(II) in drinking water samples. A comparative evaluation of ELISA and KinExA revealed that KinExA had a higher sensitivity and better precision than ELISA, whereas both assays had comparable accuracy. Both ELISA and KinExA were superior to the existing atomic spectrometric methods for Cu(II) in terms of sensitivity, convenience, and analysis throughputs. The proposed ELISA and KinExA are anticipated to effectively contribute to assessing Cu(II) concentrations and control the exposure of humans to its potential toxicities.

## 1. Introduction

Copper is an essential trace element for the life of both mammals and plants. It plays an important role in the function of several enzymes controlling carbohydrate and lipid metabolism. Dietary copper comes from organ meats, green leafy vegetables, some fruits, and nuts. Drinking water is considered as a potential major source of copper intake [1,2]. A high copper concentration exists in drinking water because of the widespread use of copper pipes in household plumbing in some countries. The copper content of drinking water is highly variable and is influenced by the natural mineral content and pH of water. In addition, copper salts are added in some countries to drinking water to control the growth of algae [3,4]. These practices mean that human exposure to high doses of copper from drinking water is expected. Human exposure to copper overload is associated with acute gastrointestinal upset [5,6], severe liver necrosis, hepatic failure and carcinogenesis, culminating in Wilson’s disease [7], Indian childhood cirrhosis [8,9], and idiopathic toxicosis [10]. The presence of high concentrations of copper ions in vivo can change the redox system of cells causing toxicity [11] and damaging DNA, proteins, and lipids [12]. High concentrations of copper ions can non-specifically react with some amino acid residues, leading to misfolding of proteins’ structures [13]. Free copper ions also compete with other substances involved in the functions of some enzymes, resulting in disrupting their normal functions [14]. In addition, the accumulation of copper in the human body contributes to atherosclerosis and rapid cell aging, posing a huge concern to public health [15,16,17]. Furthermore, excretion of copper from livestock causes soil deterioration and water pollution, which can negatively affect the growth rate of plants [18,19,20,21] and pose ecological concerns [22,23].

Hygienists and public authorities have a primary objective of reducing human exposure to copper. To achieve this, various efforts have been undertaken to determine a safe concentration of copper in drinking water, establishing health-based safety guidelines and maximum allowable limits in human serum. These guidelines have been made mandatory through legislation in multiple countries and are endorsed by the World Health Organization (WHO).

In accordance with the Safe Drinking Water Act (Section 1412), the U.S. Environmental Protection Agency (EPA) was mandated to set and publish maximum contaminant-level goals, as well as to establish national primary drinking water regulations for substances that could potentially harm human health. The Food and Nutrition Board (FNB) has recommended a daily dietary intake of copper for adults, ranging from 1.5 to 3.0 mg/day. The EPA has set a maximum contaminant-level goal for copper in drinking water at 1.3 mg/L, with the aim of safeguarding against adverse effects on the gastrointestinal tract. The WHO has proposed a provisional drinking water guideline of 2 mg/L for copper. This guideline was issued by the Joint Food and Agriculture Organization (FAO)/WHO Expert Committee on Food Additives. The WHO assumes that a 60 kg adult consumes 2 L of water per day, with 10% of the maximum tolerable daily intake attributed to drinking water [24].

In light of these health issues, numerous health authorities at the governmental level have urged researchers and administrators to conduct extensive investigations into the impact of copper in drinking water on human well-being. The presence of accurate and reliable analytical methods that are capable of delivering timely data on copper concentrations in drinking water samples could potentially lower the occurrence of copper poisoning in individuals. Different analytical techniques and methods are recommended by the EPA for drinking water compliance monitoring of copper and other secondary contaminants [25,26,27]. Of the methods, inductively coupled plasma atomic emission spectroscopy (ICP-AES) is the most commonly employed. This technique usually provides reasonable sensitivity; however, the analysis usually needs a large sample volume to ensure adequate sensitivity. Also, the number of samples that can be analyzed is limited by the fact that samples are analyzed one at a time. In addition, the instrument is expensive and requires highly qualified or skilled personnel to operate and conduct the entire analytical process. Therefore, the sample turnaround time is relatively slow, and the method is not adaptable to screening of large number of samples.

Immunoassays are a better alternative for elemental analysis. Local and governmental agencies find this approach appealing due to its notable advantages over conventional methods. These methods offer rapid results, are easily executed, portable to the analysis site, require minimal sample preparation, and enable high throughput. Additionally, research has indicated that the utilization of immunoassays can lead to a reduction in analysis costs by 50% or more [28]. Immunoassays were successfully established in recent years for metal ions as far more suitable antibodies could be generated. The generation of antibodies for metal ions involved the use of bifunctional chelating agents for metal ions and the resulting metal chelates could be covalently linked to carrier proteins. The metal chelate-protein conjugates showed distinct unique antigenic properties which could be used to generate monoclonal antibodies directed to metal ions [29,30]. Microwell-based enzyme-linked immunosorbent assays (ELISA) and kinetic exclusion assays (KinExA) employing a KinExA^TM^ immunosensor are dominant immunoassays as they offer several advantages, such as a convenient automated analysis; thus, they are considered as high-throughput techniques. In a previous study [31], an ELISA has been described for the determination of copper ions in drinking water; however, the assay lacked the specificity for trace monitoring of copper contents in drinking water because cobalt demonstrated high cross reactivity (26–31%) with copper ions. KinExA has not been described yet for copper ions in drinking water.

The present study describes the generation of a new monoclonal antibody that could recognize the Cu(II)-EDTA complex, but not free EDTA, and detected Cu(II) without interference from many other metal ions in drinking water samples. The monoclonal antibody, designated as 8D66, was subsequently utilized in the development of new ELISA and KinExA assays with a high sensitivity and specificity for the trace determination of Cu(II) in drinking water samples at concentrations as low as 0.5 and 0.1 ng/mL for ELISA and KinExA, respectively.

## 2. Results and Discussion

### 2.1. Immunization and Immune Response

**Figure 1:**
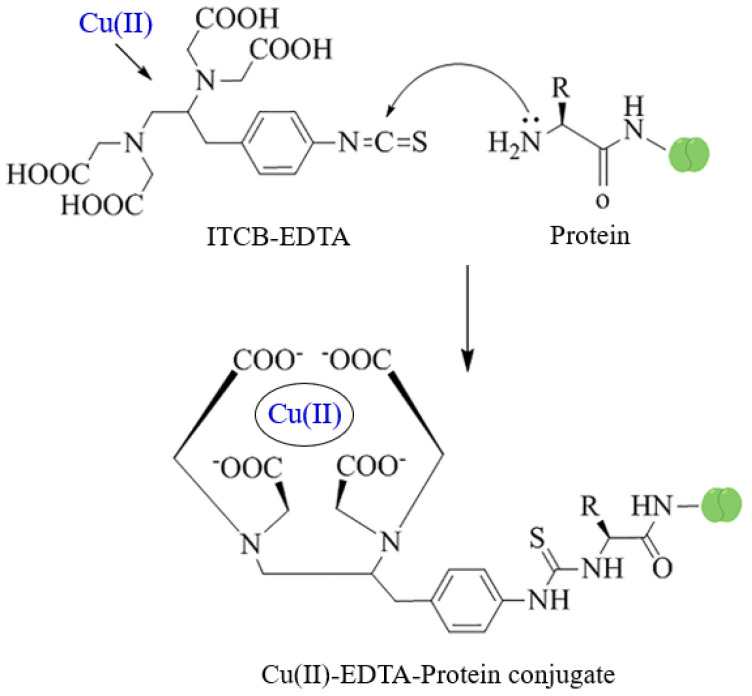
Preparation of Cu(II)-EDTA-Protein conjugates. ITCB-EDTA was 1-(4-Isothiocyanobenzyl)-ethylenediamine N,N,N′,N′-tetraacetic acid. Protein was BSA and KLH.

**Figure 2:**
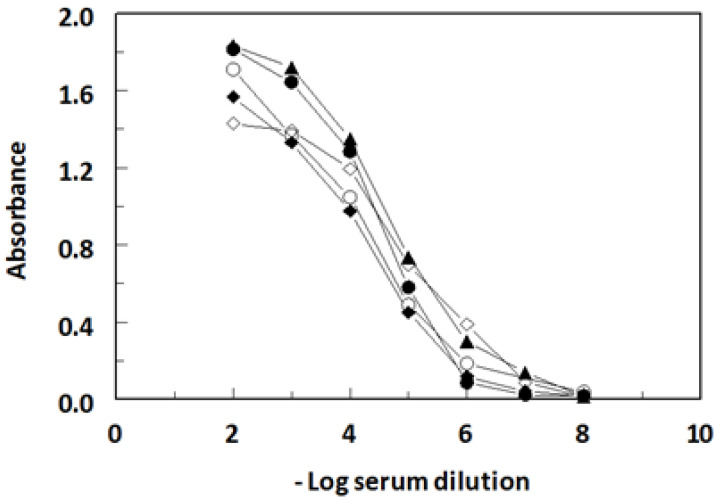
Titration curves of serum from BALB/c mice immunized with Cu(II)-EDTA-KLH. Blood was collected from tail vein of each mouse, and the resulting serum was serially diluted into HBS. The titration was performed by direct ELISA. The titer of polyclonal antibody response to immobilized Cu(II)-EDTA-BSA was defined as the serum dilution at approximately halfway between the maximum and minimum absorbance in the titration curve. The serum titers for mouse 1 (⬤), 2 (◯), 3 (▲), 4 (◆), and 5 (◊) were 40,000, 20,000, 50,000, 20,000, and 100,000, respectively.

**Figure 3:**
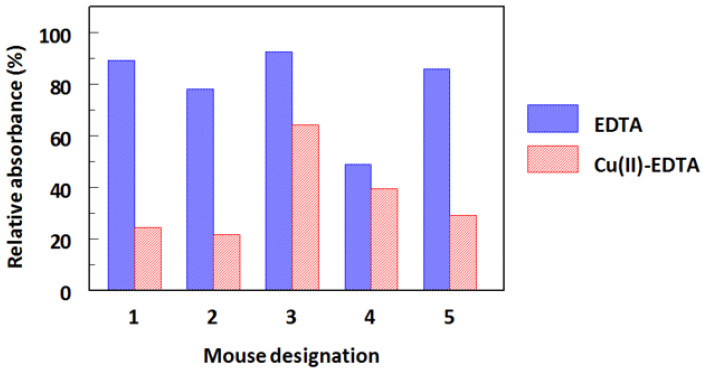
Relative recognizing specificity of serum from BALB/c mice immunized with Cu(II)-EDTA-KLH to metal-free EDTA and Cu(II)-EDTA complexes. Serum dilutions that gave 1 absorbance unit in the titration curves were prepared in HBS and used for competitive ELISA. Aliquots of each serum were mixed with HBS amended with 5 mM of metal-free EDTA or 5 mM EDTA and 50 ng/mL of Cu(II). The mixtures were dispensed into microwells of ELISA plates coated with Cu(II)-EDTA-BSA, and the assay was manipulated as described in the Experimental Section. Serum dilutions used for mice 1, 2, 3, 4, and 5 were 30,000 (mouse 1, 3, and 5) and 10,000 (mouse 2 and 4).

The Cu(II)-EDTA complex is a low molecular weight non-immunogenic hapten, thus it is unable to induce an immune response in animals. Therefore, a bifunctional EDTA derivative (ITCB-EDTA) was complexed with Cu(II) and the complex was covalently linked to KLH, a large immunogenic protein (Figure 1). The resulting Cu(II)-EDTA-KLH conjugate was used for immunization of BALB/c mice by multiple injections into the peritoneal cavity of mice. The immune response of mice and their induction of antibodies recognizing the Cu(II)-EDTA complex was assessed by non-competitive ELISA. As shown in Figure 2, sera from all mice recognized and bound to the coated Cu(II)-EDTA-BSA conjugate. These data revealed that an adequate immunization of all animals was attained. In order to select the most appropriate mouse for use in hybridoma construction, the specificity of these five sera to both the EDTA and Cu(II)-EDTA complex was determined by competitive ELISA. As shown in Figure 3, all sera showed a preferential binding to Cu(II)-EDTA more than EDTA. Mouse 4 showed comparable binding to both EDTA and Cu(II)-EDTA. Mouse 1 was given an additional intraperitoneal booster injection with Cu(II)-EDTA-KLH, and its spleen cells were used for hybridization with myeloma cells.

### 2.2. Hybridoma Screening

**Figure 4:**
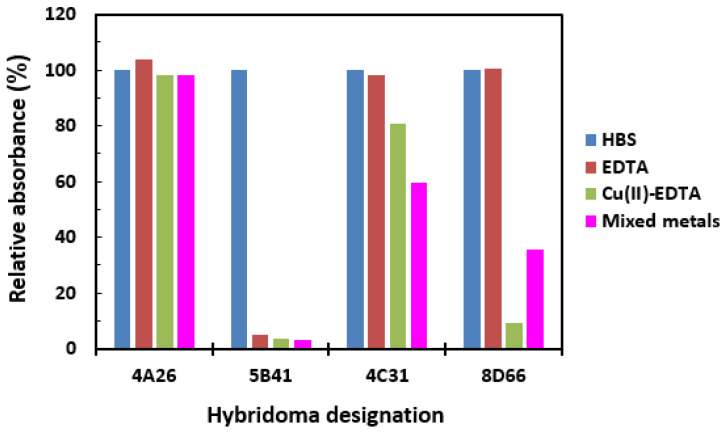
Representative results of hybridoma screening by competitive ELISA. Hybridoma culture supernatants were diluted with HBS and mixed with metal-free EDTA, Cu(II)-EDTA (Cu(II) mixed with EDTA), or mixed metals (prepared in EDTA) to yield a final EDTA concentration of 5 mM and a final metal concentration of 200 ng/mL. The mixtures were dispensed into microwells coated with Cu(II)-EDTA-BSA, and the assay was completed as usual. Mixed metals solution was a mixture of equal concentrations (20 ng/mL) of Cd(II), Pb(II), Hg(II), Mn(II), Mg(II), Fe(III), Co(II), Ni(II), Zn(II), and Ca(II).

The hybridomas (582 clones) resulting from the fusion with myeloma cells underwent an initial screening using a direct ELISA to identify antibodies capable of binding to Cu(II)-EDTA-BSA. From this screening, 42 positive clones were obtained. These clones were then subjected to a competitive ELISA to determine their specificity. Supernatants from these 39 clones were tested to assess their ability to bind to immobilized Cu(II)-EDTA-BSA in the presence of free binding competitors. The results from these 39 clones could be categorized into four types, represented by four distinct hybridomas: 4A26, 5B41, 4C31, and 8D66 (Figure 4). Eighteen clones exhibited a binding response similar to that of clone 4A26. However, the antibodies produced by hybridoma 4A26 demonstrated binding to Cu(II)-EDTA-BSA that could not be inhibited by 5 mM EDTA, 200 µg/mL Cu(II) in 5 mM EDTA, or 200 µg/mL of mixed metals. The mixed metals solution comprised 20 µg/mL of each of Cd(II), Pb(II), Hg(II), Mn(II), Mg(II), Fe(III), Co(II), Ni(II), Zn(II), and Ca(II) in 5 mM EDTA. Consequently, it was concluded that these clones primarily generated antibodies recognizing the benzyl group present in the linker region between the EDTA and BSA proteins of the immobilized Cu(II)-EDTA-BSA conjugate, and thus, these clones were excluded from further characterization. Fifteen clones exhibited responses similar to that of clone 5B41. The antibodies produced by these clones were strongly inhibited by 5 mM EDTA, Cu(II)-EDTA, and mixed metals-EDTA complexes. It appeared that the antibodies produced by these clones primarily targeted EDTA. These clones were further tested for isolation of a monoclonal antibody specific to EDTA rather than Cu(II)-EDTA. The isolation binding properties of this monoclonal antibody will be published elsewhere. Six clones showed binding characteristics similar to that of the clone designated 4C31. The antibodies secreted by these clones were slightly inhibited by Cu(II)-EDTA and mixed metals-EDTA, therefore they were excluded from further testing. The remaining three clones showed characteristics similar to that exemplified by 8D66. The antibodies secreted by these two clones were strongly inhibited by Cu(II)-EDTA, relative to that exhibited by the mixed metals. Clone 8D66 was chosen for further experiments since this clone showed a higher specificity to Cu(II)-EDTA when other individual metals were tested.

Clone 8D66 was subcloned by limiting dilution to assure that it is monoclonal. The 8D66 hybridoma secreted a monoclonal antibody of IgG1 subtype with a kappa light chain. This clone was grown in a CL1000 hybridoma culture device to obtain a high antibody concentration in the culture media. The 8D66 monoclonal antibody was purified and its IgG1 content was found to be 0.6 mg/mL with a specific titer of 460,000/mg of IgG1 when tested by non-competitive ELISA, as described in the Experimental Part.

### 2.3. Antibody Specificity

Because metals are commonly encountered in drinking water, it was important to determine the specificity of the 8D66 antibody for Cu(II) among the other metal ions encountered in drinking water. The potential interference by individual metal ions was examined over a wide range of concentrations. The metal ions examined were Zn(II), Ni(II), Hg(II), Cd(II), Pb(II), Mn(II), Mg(II), Fe(III), and Ca(II). All these metal ions were transformed to their EDTA complexes, and their solutions were analyzed by competitive ELISA and KinExA. IC_50_ is defined as the concentration required to cause 50% of the antibody binding to immobilized Cu(II)-EDTA-BSA. The cross reactivity was calculated as the IC_50_ of Cu(II)/IC_50_ of metal ion, expressed as a percentage. Table 1 shows the cross reactivity of different metal ions in the assay. The presence of metal ions most commonly present at relatively high concentrations in drinking water samples, Ca(II), Mg(II), and Fe(III), did not interfere with the Cu(II) assay at concentrations as high as 200 μg/mL. A low cross reactivity was observed only with Zn(II), Ni(II), and Hg(II): the metal ions present, if any, at low concentrations in drinking water. The results obtained by ELISA are given in Table 1, and similar cross reactivity (%) values were obtained by KinExA. These results indicated the adequate specificity of the present method for the analysis of Cu(II).

### 2.4. Description of ELISA and KinExA

**Figure 5:**
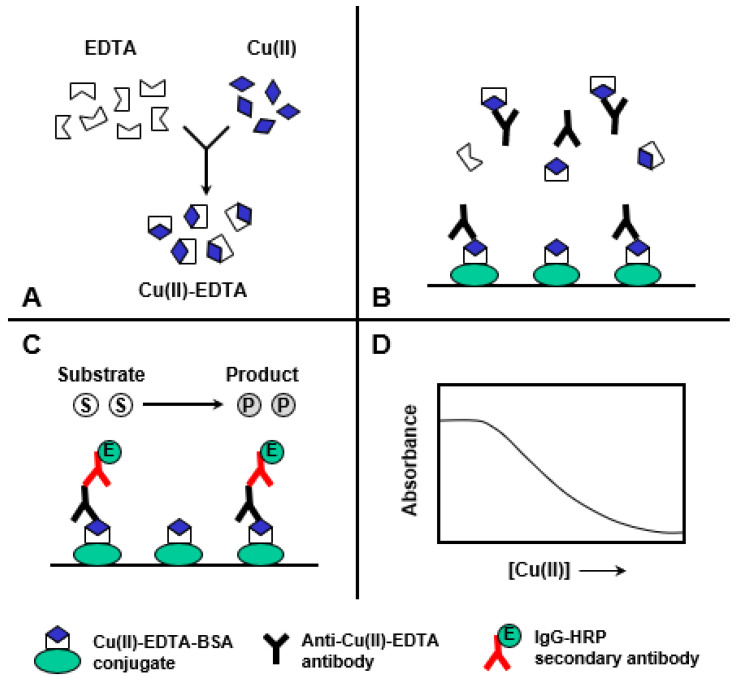
Schematic diagram of the proposed ELISA for Cu(II). (**A**): formation of Cu(II)-EDTA complexes, (**B**): competitive binding reaction, (**C**): color development, and (**D**): measurement and generation of binding calibration curve.

**Figure 6:**
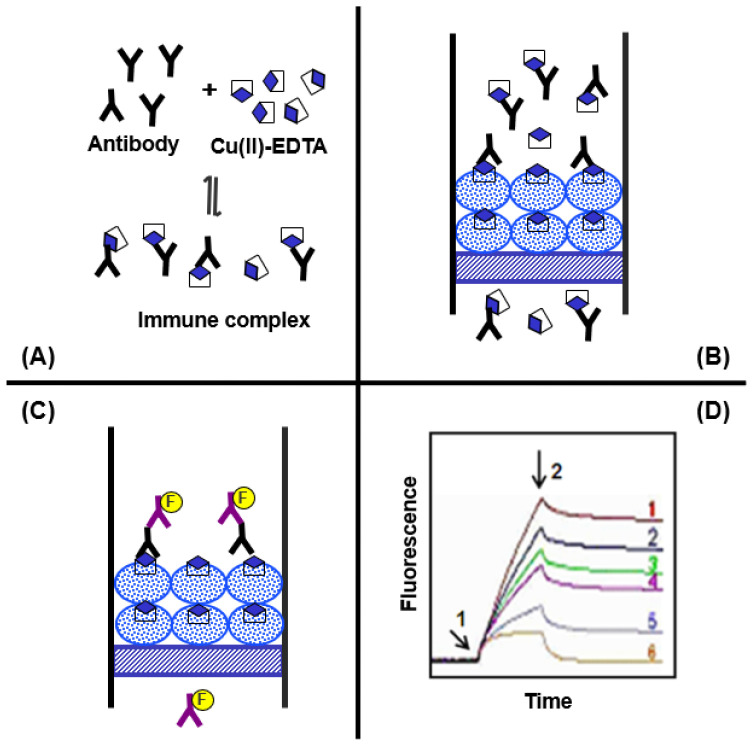
Schematic presentation of operational steps of KinExA for Cu(II). (**A**): formation of Cu(II)-EDTA complexes, (**B**): passing immune complexes through PMMA beads, (**C**): passing fluorescent labeled IgG-FITC through the beads, and (**D**): generation of KinExAgrams.

This study describes the development of ELISA and KinExA for the trace determination of Cu(II) in drinking water samples. The features and steps of ELISA and KinExA are illustrated in Figure 5 and Figure 6, respectively.

ELISA involved four main steps (A–D), shown in Figure 5A: The sample containing Cu(II) is diluted with a buffer containing a molar excess of metal-free EDTA to form Cu(II)-EDTA complexes. (B) The solution containing the excess EDTA and Cu(II)-EDTA complexes is mixed with an antibody in the microwell of ELISA plates coated with the Cu(II)-EDTA-BSA conjugate. The Cu(II)-EDTA complex, in the solution, competes with the immobilized Cu(II)-EDTA-BSA conjugate for a limited amount of antibody binding sites. (C) After a wash step, IgG-HRP is added and allowed to bind to the anti-Cu(II)-EDTA antibody. A second wash step is applied to remove any unbound IgG-HRP, and the color is developed by the addition of colorimetric TMB substrate. (D) Absorbances are measured and plotted as the corresponding Cu(II) concentrations. The four-parameter equation fit of the data is used for calculating the Cu(II) concentrations in the sample solutions.

KinExA is an automated flow fluorescence competitive assay, and the setup and operation details of the KinExA™ 3200 immunosensor have been previously outlined in a study [32]. In the KinExA method (Figure 6), (A) a mixture of Cu(II)-EDTA and its specific antibody is allowed to reach equilibrium, forming an immune complex. (B) The instrument automatically draws the immune complex solution and rapidly passes it through a flow cell containing PMMA beads coated with a Cu(II)-EDTA-BSA conjugate. During the flow, the free antibody molecules bind to the immobilized Cu(II)-EDTA-BSA, while those bound to the free Cu(II)-EDTA in solution do not. Since the flow is swift, there is minimal dissociation of the immune complex. Consequently, the anti-Cu(II)-EDTA antibody molecules bound to the immobilized Cu(II)-EDTA-BSA are kinetically excluded from binding. (C) The quantity of anti-Cu(II)-EDTA antibody captured by the immobilized Cu(II)-EDTA-BSA is then quantified using IgG-FITC as the second antibody. The fluorescence intensity is continuously monitored by the photodiode of the KinExA™ 3200 system hardware. (D) Fluorescence time curves (KinExAgrams) are generated. Curve 1 corresponds to a Cu(II) concentration of zero, and curve 6 corresponds to a saturating Cu(II) concentration. Curves 2–5 represent Cu(II) concentrations ranging from zero to saturation. Arrow 1 indicates the introduction of IgG-FITC, while arrow 2 indicates the cessation of its introduction and the initiation of a second buffer wash. The net fluorescence intensities measured on the PMMA beads coated with Cu(II)-EDTA-BSA are inversely correlated with the Cu(II) concentrations in the original samples.

### 2.5. Optimization of Reagent Concentrations for Development of ELISA

Optimal concentrations of the Cu(II)-EDTA-BSA conjugate required for immobilization onto the microwell plate and the best working concentration of the 8D66 monoclonal antibody were determined by a checkerboard titration immunoassay, using varying concentrations of both the conjugate and purified antibody. The conjugate and antibody concentrations that gave 0.8–1.2 absorbance units were considered as optimum binding conditions. The optimum concentrations were 0.5 and 0.25 μg/mL for the Cu(II)-EDTA-BSA conjugate and 8D66 antibody, respectively. These concentrations were used in all subsequent experiments.

**Figure 7:**
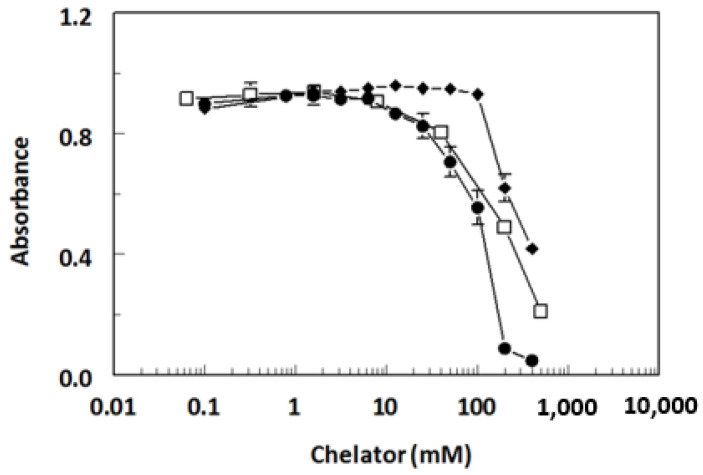
Binding of 8D66 monoclonal antibody to metal ion chelators. Competitive ELISA was performed using metal-free chelator diluted into HBS. Data are shown for EDTA (⬤), CDTA (☐), and DTPA (◆). Determinations were performed in duplicate, and values are plotted ± SD.

**Figure 8:**
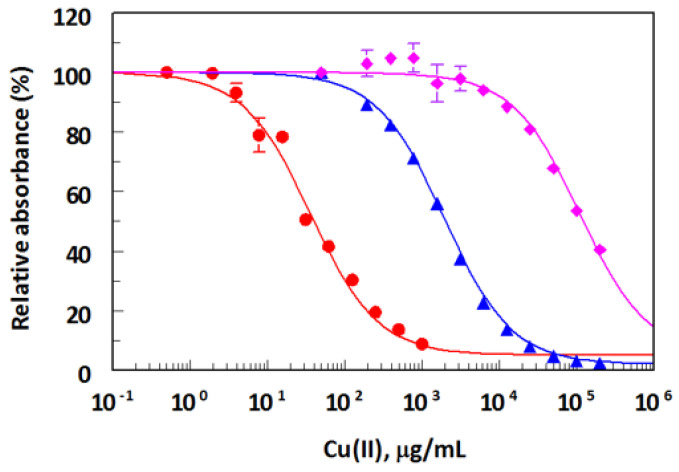
Effect of the structure of metal ion chelator on the sensitivity of ELISA for Cu(II). Competitive ELISA was performed using atomic absorption grade Cu(II) diluted into HBS amended with 10 mM metal-free EDTA (⬤), CDTA (▲), or DTPA (◆). Determinations were performed in duplicate, and values are plotted ± SD.

To determine the effect of a chelator structure and its concentrations on the assay, three different chelators were tested by competitive assay: EDTA, CDTA, and DTPA. The assay was insensitive to metal-free chelator concentrations as high as 10 mM for EDTA and CDTA, and 100 mM for DTPA (Figure 7). The sensitivity of the Cu(II) assay was very dependent on the structure of the chelator; the most sensitive assay was obtained when EDTA was used as chelator (Figure 8). A 10 mM EDTA was used for all subsequent experiments.

The other experimental conditions were also optimized, and the optimum values are given in Table 2.

### 2.6. Optimization of KinExA Conditions and Its Running

**Figure 9:**
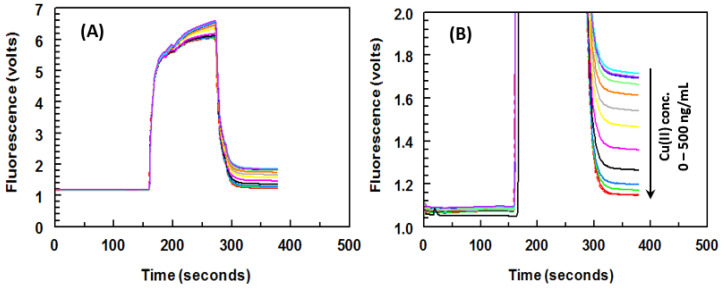
Panel (**A**): real raw trend-line fluorescence responses (KinExAgram) obtained by the KinExA^TM^ 3200 instrument for varying concentrations of Cu(II) (0–500 ng/mL). Panel (**B**): the same signals; however, they are presented on a different scale for the fluorescence (volts). All Cu(II) concentrations were prepared in HBS containing EDTA (10 mM).

A series of experiments were conducted to determine the optimal concentrations of the Cu(II)EDTA-BSA conjugate for coating onto PMMA beads and the 8D66 antibody for effective competitive binding reactions. The concentrations that resulted in the lowest IC50 value were considered optimal. The optimal concentrations were found to be 1 μg/mL for Cu(II)-EDTA-BSA and 0.5 μg/mL for the 8D66 antibody. The best coating of Cu(II)-EDTA-BSA onto PMMA beads was achieved with a coating time of 2 h at 37 °C. Other experimental conditions were also optimized, and their optimal values are provided in Table 2. Under these optimized conditions, a KinExAgram (a representation of instrument response over time) was generated for various concentrations of Cu(II), ranging from 0.02 to 5 ng/mL (Figure 9). The segment of the KinExAgram from 0 to 165 s corresponds to background signals generated during the flow of the immune complex solution through the PMMA beads in the instrument’s flow cell. The segment from 166 to 280 s represents the flow of IgG-FITC through the beads and its capture by the 8D66 antibody, which has already been captured by Cu(II)-EDTA-BSA coated onto the PMMA beads. This process is followed by a washing step from 281 to 385 s to remove any excess IgG-FITC molecules trapped on the beads. Consequently, the top curve in the KinExAgram represents the blank solution (0 concentration of Cu(II)), while the bottom curve corresponds to the highest concentration of Cu(II) (5 ng/mL).

### 2.7. Validation of ELISA and KinExA

#### 2.7.1. Calibration Curves and Detection Limits

**Figure 10:**
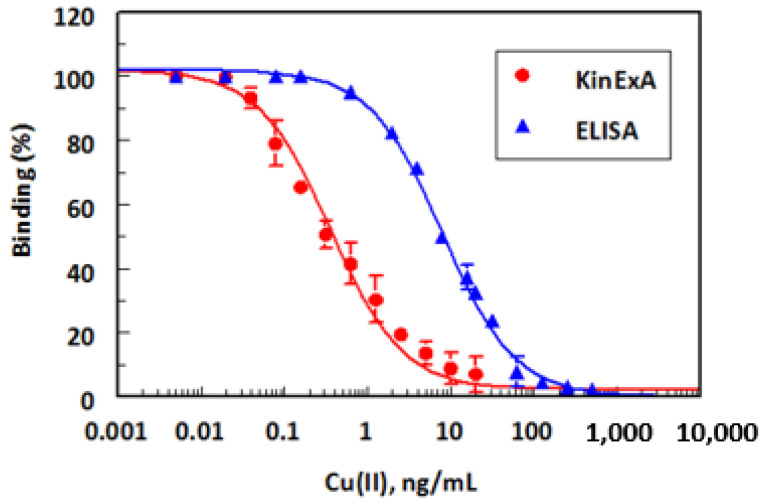
Calibration curves for determination of Cu(II) in drinking water by ELISA and KinExA. Values plotted are mean ± SD of 5 and 3 determinations in ELISA and KinExA, respectively.

The calibration curve was generated using atomic absorption grade Cu(II) at concentration ranges of 0.002–500 and 0.002–100 ng/mL for ELISA and KinExA, respectively (Figure 10). The limits of detection, defined as the lowest measurable concentration of Cu(II) that could be distinguishable from zero concentration ±3 SD, were determined. Based on the basis of three replicate measurements, the limits of detection (LOD) were 0.1 and 0.02 ng/mL for ELISA and KinExA, respectively. It is wise to mention that the achieved LOD values for both ELISA and KinExA are much lower than the maximum allowable concentrations of Cu(II) in drinking water (2 µg/mL) which are recommended by FNB, EPA, and WHO [24].

#### 2.7.2. Precision and Recovery

The within- and between-assay precision was determined at three different levels of Cu(II) concentrations (Table 3). Both ELISA and KinExA gave satisfactory results over all the tested levels of concentration; the coefficients of variations did not exceed 6.8 and 6.2% for ELISA and KinExA, respectively (Table 3).

Analytical recovery studies were performed by adding various known amounts of atomic absorption grade Cu(II) to drinking water samples. Each sample was subsequently treated and analyzed for Cu(II) content by both ELISA and KinExA, as described in the Experimental Section. The determined concentrations were treated according to the following equation to calculate the resulting percent recovery: percent recovery = drinking water sample concentration/buffer sample concentration × 100. The mean analytical recovery was 100.9 ± 3.5 and 100.6 ± 3.1% for ELISA and KinExA, respectively (Table 4).

### 2.8. Comparison of ELISA and KinExA with ICP-AES

**Figure 11:**
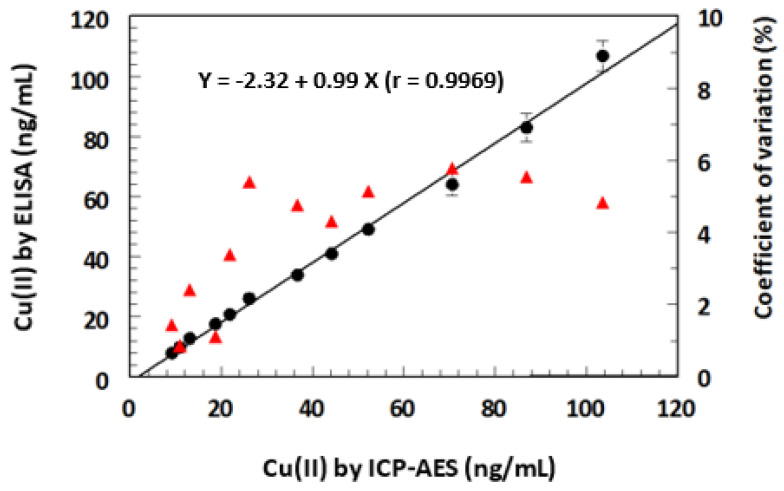
Comparison of ELISA with inductively coupled plasma atomic emission spectroscopy (ICP-AES) for analysis of drinking water samples spiked with Cu(II). A series of Cu(II)-spiked samples were prepared in the concentration range 5–100 ng/mL. The samples were analyzed by ELISA and IC-PAES. The values of Cu(II) found by ELISA (⬤) plotted are mean ± SD of 3 determinations. Coefficients of variations (▲) were calculated as SD/mean, expressed as percentages. Fitting equation and its correlation coefficient (r) are given on the graph.

In a series of experiments, drinking water samples were spiked with varying concentrations of Cu(II), and the samples were independently analyzed for their Cu(II) contents by ELISA, KinExA, and inductively coupled plasma atomic emission spectrometry (ICP-AES). The sensitivity of both ELISA and KinExA was superior to that of ICP-AES; therefore, the concentrations of the samples subjected to the comparative study were in the dynamic range of ICP-AES. These samples were diluted to make their concentrations within the dynamic ranges of both ELISA and KinExA. The results obtained by ELISA and KinExA were plotted as a function of those measured by ICP-AES. Figure 11 shows the results of ELISA, and a similar profile was obtained with KinExA; it was almost superimposed on that of the ELISA. For clear presentation of the data, the results of only ELISA are presented in the figure. The results were statistically compared, and it was found that the concentrations measured by both ELISA and KinExA correlated very well with those measured by ICP-AES. A linear regression analysis of the results yielded linear equations:

For ELISA: Y = −2.32 + 0.99 × (r = 0.9969)

For KinExA: Y = 1.85 + 1.04 × (r = 0.9982)

where Y is the Cu(II) concentration measured by ELISA or KinExA, X is the Cu(II) concentration measured by ICP-AES, and r is the correlation coefficient of the linear regression analysis. The high values of the correlation coefficient (r), slopes (approximately 1), and low coefficient of variations (<6%) revealed the reliability (high accuracy and precision) of the results obtained by ELISA and KinExA. In addition, the results confirmed the successful applicability of both ELISA and KinExA for the determination of Cu(II) in drinking water.

### 2.9. Comparative Evaluation of ELISA and KinExA

A comparative assessment was conducted to evaluate the analytical performance of ELISA and KinExA. The findings demonstrated that KinExA exhibited superior sensitivity, with a lower limit of detection (LOD) compared to ELISA. This improved sensitivity of KinExA can be attributed to the larger surface area of the PMMA beads used in the assay compared to the microwells of an ELISA plate. The surface area of PMMA beads is approximately 260 mm^2^, whereas each microwell in the ELISA format is only 64 mm^2^ [33]. The increased surface area of the PMMA beads allowed for the capture of a higher number of 8D66 antibody molecules, leading to enhanced fluorescence signals. Furthermore, the short duration in which the 8D66 antibody passed through the capture reagent (immobilized Cu(II)-EDTA-BSA) in the KinExA assay minimized the competitive binding capacity of Cu(II)-EDTA-BSA. Both ELISA and KinExA exhibited comparable accuracies, as evidenced by recovery values ranging from 96.4% to 105.2% for ELISA and 96.7% to 103.5% for KinExA.

In terms of precision, KinExA demonstrated superior precision compared to ELISA, as evidenced by the lower coefficients of variation. KinExA achieved better precision due to its utilization of PMMA beads with a higher surface area. Consequently, the precision of KinExA relied solely on the concentrations of the 8D66 and IgG-FITC antibodies, which were automatically drawn by the KinExA instrument with a high level of precision. On the other hand, in ELISA, precision primarily depended on the uniformity of the quantity of Cu(II-EDTA-BSA immobilized onto the microwells of the ELISA plates. Since these quantities are manually transferred to the wells, slight variations in uniformity may occur, leading to differences in assay precision.

In KinExA, the reagents passed through PMMA beads with a high flow rate and, accordingly, the mass transport limitation encountered in ELISA has been minimized [34,35].

## 3. Experimental

### 3.1. Instruments

EL×800 absorbance microplate reader and ELx 50 automatic microplate strip washer were products of Bio-Tek Instruments Inc. (Winooski, VT, USA). KinExA™ 3200 immunosensor was obtained from Sapidyne Instruments Inc. (Boise, ID, USA). Model MINI/18 Incubator was obtained from Genlab Ltd. (Widnes, UK). Milli-Q water purification system was obtained from Labo, Millipore Ltd. (Bedford, NY, USA). UV-1601 PC double beam spectrophotometer was from Shimadzu (Kyoto, Japan). Nutating mixer was from Taitec (Saitama-ken, Japan).

### 3.2. Materials

Atomic absorption spectroscopy standard metals (1000 mg/L in 2% HNO_3_) were obtained from Perkin-Elmer Co. (Norwalk, CT, USA). 1-(4-Isothiocyanobenzyl)-ethylenediamine N,N,N′,N′-tetraacetic acid (ITCB-EDTA) was purchased from Dojindo Laboratories (Gaithersburg, MD, USA). Ultrapure bovine serum albumin (BSA) and an IsoStrip mouse monoclonal antibody isotyping kit were purchased from Boehringer Mannheim Biochemicals (Indianapolis, IN, USA). Keyhole limpet hemocyanin (KLH) was obtained from Calbiochem-Novabiochem Corporation (La Jolla, CA, USA). BALB/c mice (6-week-old females) were purchased from Charles River Laboratories (Wilmington, MA, USA). SP2/0-Ag14 myeloma cells were obtained from the American Type Culture Collection (Rockville, MD, USA). ClonaCell-HY hybridoma cloning kit was purchased from Stemcell Technologies Inc. (Vancouver, Canada) and used according to the manufacturer’s instructions. CELLine CL1000 device for culture of suspension cells was obtained from Integra Biosciences Inc. (Ijamsville, MD, USA). Ethylenediaminetetraacetic acid (EDTA), diaminocyclohexanetetraacetic acid (CDTA), diethylenetriaminepentaacetic acid (DTPA), goat anti-mouse IgG (Fc specific) conjugated to horseradish peroxidase (IgG-HRP), goat anti-mouse IgG conjugated with fluorescein isothiocyanate (IgG-FITC), and Freund’s adjuvants (complete and incomplete) were purchased from Sigma-Aldrich Chemical Co. (St. Louis, MO, USA). Immobilized protein G resin and BCA protein assay kit were purchased from Pierce Chemical Co. (Rockford, IL, USA) and used according to the manufacturer’s directions. Centricon-30 filter was from Amicon, Inc. (Beverly, MA, USA). ELISA high-binding microwell plates were a product of Corning/Costar, Inc. (Cambridge, MA, USA). 3,3′,5,5′-Tetramethylbenzidine (TMB) peroxidase substrate was obtained from Kirkegaard-Perry Laboratories (Gaithersburg, MD, USA). Polymethylmethacrylate (PMMA) beads (140–170 mesh, 98 μm) were acquired from Sapidyne Instruments Inc. (Boise, ID, USA). Metal-free disposable pipette tips were a product of Oxford Labware, Inc. (St. Louis, MO, USA). All glassware was washed with mixed HCl/HNO_3_ acids and liberally rinsed with purified deionized water. All plastic wares were soaked overnight in 3 M HCl and rinsed with purified deionized water before use.

### 3.3. Preparation of Copper-EDTA-Protein Conjugates

Protein conjugates of Cu(II)-EDTA with BSA and KLH were prepared by a modification of the method previously reported by Chakrabarti et al. [36]. Briefly, a solution of ITCBE (2.7 mM, in 0.1 M sodium phosphate buffer, pH 9.5) was prepared. Aliquot (1 mL) of ITCBE solution was mixed with equal volume of copper nitrate solution (2.7 mM, in 50 mM phosphate buffer, pH 9.5). Protein (BSA and KLH) solution (2 mL, 20 mg/mL, in 0.1 M sodium phosphate buffer, pH 9.5) was added. The pH of the reaction mixture was maintained at pH 9.5 for 30 min by addition of KOH when necessary. The reactions were stirred overnight at 25 °C. The unreacted low molecular weight components (Cu(II) and EDTA) were removed by buffer exchange using a Centricon-30 filter that had been treated with 100 mM EDTA solution and liberally rinsed with water before use. Protein concentrations of the conjugates were determined using BCA kit, and the extent of substitution of free amino groups on the protein was determined by estimation of free amino groups before and after the conjugation reaction [37]. The extents of conjugation of BSA and KLH were 20.6% and 77.8% for their Cu(II)-EDTA conjugates, respectively.

### 3.4. Immunization and Cell Fusion

Five mice were injected intraperitoneally with 50 μg of Cu(II)-EDTA-KLH conjugate emulsified in Freund’s adjuvant. The second, third, and fourth injections were conducted after 15, 21, and 28 days from the first injection. Three days after the fourth boost, blood was collected from the tail vein, and the antibody induction was tested by non-competitive screening ELISA (described below). The mouse showing the highest immune response to Cu(II)-EDTA was given a final boost by intraperitoneal injection of 50 μg of Cu(II)-EDTA-KLH conjugate in phosphate-buffered saline (PBS; 137 mM NaCl, 3 mM KCl, 10 mM sodium phosphate buffer, pH 7.4). After 4 days, spleen cells of the mouse were harvested and fused with SP2/0-Ag14 myeloma cells, and the cells were cultivated in 96-well tissue culture plates. Culture supernatants were collected from viable, proliferating hybridomas, and screened for productions of antibodies by competitive ELISA (described below) using Cu(II)-EDTA-BSA immobilized onto wells of ELISA plate and Cu(II)-EDTA as a competitor (antibody binding inhibitor). Hybridomas secreting antibodies specific to Cu(II)-EDTA were subcloned two times by limiting dilution. The most appropriate clone was expanded in CELLine culture device to obtain high concentration of antibody. Supernatant harvest and nutrient medium exchange were performed according to the manufacturer’s instructions. IgG content of the culture supernatant was isolated by using protein G column. The type and isotype of monoclonal antibody were determined by IsoStrip antibody isotyping kit, according to the manufacturer’s instructions. Protein concentration of the purified antibody was determined by Bradford method [38].

### 3.5. Non-Competitive and Competitive ELISA for Screening

The non-competitive ELISA procedure can be summarized as follows: Cu(II)-EDTA-BSA conjugate (2 µg/mL) was coated onto ELISA plates and incubated at 37 °C for 2 h. Subsequently, the plates were blocked with BSA (3%, *w*/*v*) at 37 °C for 1 h. The Cu(II)-EDTA-BSA conjugate and BSA used for coating and blocking were prepared in a HEPES-buffered saline solution (HBS) with a pH of 7.4, containing 137 mM NaCl, 3 mM KCl, and 10 mM HEPES. Mice sera or hybridoma culture supernatants were diluted with HBS, and 100 µL aliquots of the diluted samples were added to the wells of the coated plates. The plates were then incubated at 37 °C for 2 h. After the incubation, the plates were washed three times with PBST solution (phosphate-buffered saline containing 0.05% Tween-20, pH 7.4). Next, 100 µL of IgG-HRP (1 µg/mL, in HBS) was added to each well, and the plates were incubated for 1 h at 37 °C. Following the incubation, the plates were washed as described above. To initiate color development, 100 µL of TMB substrate solution was added to each well, and the reaction was allowed to proceed for 15 min at room temperature (25 ± 2 °C). The color development reaction was stopped by adding 50 µL of 2 M HCl solution to each well, and the absorbance of each well was measured at 450 nm using an absorbance microplate reader.

Positive clones were screened using the competitive ELISA method, which followed a similar procedure to the non-competitive ELISA. However, in the competitive ELISA, the culture supernatants were mixed with either Cu(II)-EDTA complex or metal-free EDTA solutions before being added to the plate wells. To prepare the Cu(II)-EDTA complex, Cu(II) was added to HBS containing EDTA at a concentration of 5 mM. This solution served as the Cu(II)-EDTA complex in the competitive ELISA procedure.

### 3.6. Analysis of Cu(II) by ELISA

The ELISA plates were prepared by coating them with Cu(II)-EDTA-BSA conjugate at a concentration of 0.5 μg/mL, followed by blocking with BSA using the procedures and conditions mentioned earlier. To condition the water samples, a 10% volume of HBS containing EDTA at a concentration of 10 mM was added. Aliquots of the diluted water samples were mixed in a 1:1 ratio with a solution of 8D66 monoclonal antibody (0.25 μg/mL in HBS), and 50 μL of the mixture was added to each well of the plates. After incubating for 1 h at 37 °C, the plates were washed following the previously described method. The quantity of bound 8D66 antibody was measured using anti-mouse IgG-HRP (1 μg/mL) and TMB substrate. The color development process was conducted as outlined in the ELISA screening procedure. Standard curves for Cu(II) were generated using the same method on plates from the same series. The IC50 values were calculated using the following equation:

A = A_0_ − {(A_0_ − A_1_)[Cu(II)]/(IC_50_ + [Cu(II)])} (1)

The calculation of Cu(II) concentrations in the samples was performed using a fitting equation. In the equation, A represents the binding percentage at a specific known concentration of soluble Cu(II), A_0_ represents the binding percentage in the absence of Cu(II), A_1_ represents the binding percentage at the saturating concentration of Cu(II), and IC_50_ represents the Cu(II) concentration that causes a 50% inhibition of the binding. By applying the fitting equation, the concentrations of Cu(II) in the samples were determined.

### 3.7. Analysis of Cu(II) by KinExA

The previous report [35] provided a detailed description of the KinExA™ 3200 immunosensor and its operation. In accordance with the procedures outlined in a previous report [32], PMMA beads were coated with Cu(II)-EDTA-BSA at a concentration of 1 µg/mL, then blocked with BSA (3% *w*/*v* in HBS) and loaded into the flow cell of the instrument. For the water samples, they were prepared in HBS buffer of pH 7.4 containing 10 mM EDTA and pre-equilibrated at room temperature (25 ± 2 °C) for approximately 15 min with 8D66 antibody (0.5 µg/mL in HBS buffer, pH 7.4). The instrument withdrew 500 µL of the pre-equilibrated samples, which were then passed through the PMMA beads coated with Cu(II)-EDTA-BSA located in the flow cell of the instrument for 120 s at a flow rate of 0.25 mL/min. After the samples were run, any unbound reagents were washed-out by-passing PBS through the beads. Following this, 500 µL of anti-mouse IgG-FITC (0.25 µg/mL) was drawn and passed through the beads for 120 s at the same flow rate. Unbound IgG-FITC was subsequently removed by passing 1.5 mL of HBS through the PMMA beads for 90 s at a flow rate of 1 mL/min. The bound IgG-FITC was evaluated by measuring the fluorescence intensity. The data obtained from the KinExA Pro 20.0.1.26 software were then subjected to four-parameter curve fitting using Slide Write software, version 5.011 (Advanced Graphics Software, Inc., Rancho Santa Fe, CA, USA).

## 4. Conclusions

A monoclonal antibody (designed as 8D66) with a high selectivity for the Cu(II)-EDTA complex was generated and fully characterized. The 8D66 antibody was employed in the development and validation of microwell-based ELISA and immunosensor-based KinExA platforms for the determination of Cu(II) in drinking waters. These assays were microwell-based ELISA and immunosensor-based KinExA. Validation studies of these assays confirmed their applicability for the accurate, precise, and selective determination of Cu(II) in drinking water without interference from other commonly encountered meta ions. A comparative evaluation of ELISA and KinExA revealed that KinExA is superior to ELISA in terms of sensitivity and precision, whereas both assays have comparable accuracy. Both assays are likely anticipated to contribute to the monitoring of Cu(II) levels in drinking water in order to significantly enhance the water’s safety and decrease the risk of exposure to Cu(II)’s side effects. The use of these immunoassay systems for the determination of copper in drinking water circumvents many problems associated with the present technologies, which require sophisticated equipment in a central facility.

## Figures and Tables

**Table 1:** Specificity of Cu(II) immunoassay.

Metal Ions	IC_50_ (ng/mL) ^a^	Cross Reactivity (%) ^b^
Cu	4.7 ^c^	100 ^c^
Zn	73.4	6.4
Ni	184.2	2.6
Hg	467.8	1.0
Cd	ND ^d^	ND
Pb	ND	ND
Mn	ND	ND
Mg	ND	ND
Fe	ND	ND
Ca	ND	ND

^a^ IC_50_ is the concentration of metal-EDTA complex which inhibits the color formation in the competitive immunoassay by 50%. ^b^ Calculated as IC_50_ [metal ion]/IC_50_ [Cu(II)] × 100. ^c^ Values are mean of duplicate determinations. ^d^ Not determined because it was more than 200 µg/mL.

**Table 2:** Summary for optimum parameters and conditions of ELISA and KinExA for Cu(II) in drinking water samples.

Parameter/Condition	Optimum Value
ELISA	
Coating Cu(II)-EDTA-BSA conc. (μg/mL)	0.5
Coating buffer	HBS
Coating time (h)/temperature (°C)	2/37
BSA concentration for blocking (%, *w*/*v*)	3
Blocking with BSA: time (min)/temperature (°C)	1/37
Concentration of EDTA (mM)	10
8D66 antibody concentration (μg/mL)	0.25
IgG-HRP concentration (μg/mL)	1
Binding of IgG-HRP: time (h)/temperature (°C)	1/37
TMB: time (min)/temperature (°C)	15/25
Reagent for stopping the color reaction (M)	HCl (2)
Measuring wavelength (nm)	450
KinExA	
Coated Cu(II)-EDTA-BSA (µg/mL)	1
Volume of beads suspension (µL)	583
Flow rate of beads suspension (mL/min)	1
Time of beads drawing (s)	35
Concentration of 8D66 antibody (μg/mL)	0.5
Volume of Cu(II sample (µL)	500
Time of samples drawing (s)	120
Concentration of IgG-FITC (µg/mL)	0.25
Volume of IgG-FITC (µL)	500
Flow rate (mL/min)	0.25

**Table 3:** Precisions of the proposed ELISA and KinExA for Cu(II) at different concentration levels.

ELISA (*n* = 5)			KinExA (*n* = 3)		
Concentration (ng/mL)	Intra-Assay	Inter-Assay	Concentration (ng/mL)	Intra-Assay	Inter-Assay
0.5	5.4 ^a^	6.1	0.1	4.2	6.2
2	3.5	4.3	0.5	3.2	3.8
20	6.2	6.8	2	4.7	5.6

^a^ Values are coefficient of variation (%); mean of 3 determinations.

**Table 4:** Analytical recovery of Cu(II) added to drinking water samples and determined by the proposed ELISA and KinExA.

ELISA		KinExA	
Added (ng/mL)	Recovery ^a^ (% ± CV)	Added (ng/mL)	Recovery ^a^ (% ± CV)
1.25	105.2 ± 5.4	0.25	103.5 ± 6.2
2.5	96.4 ± 4.2	0.5	102.6 ± 5.3
5	101.8 ± 4.1	1	96.7 ± 4.9
10	98.6 ± 5.9	2	102.5 ± 4.8
20	102.6 ± 6.2	4	97.8 ± 4.2
Average	100.9 ± 3.5		100.6 ± 3.1

^a^ Values are means of 3 determinations.

## Data Availability

Data is available with the corresponding author.
